# Molecular insights into Valencene synthase (*SgTPS-V*) and its role in sesquiterpenoid biosynthesis

**DOI:** 10.1186/s12870-026-08095-8

**Published:** 2026-02-02

**Authors:** Mohammed Ali

**Affiliations:** Maryout Research Station, Genetic Resources Department, Desert Research Center, 1 Mathaf El-Matarya St., El-Matareya, Cairo, 11753 Egypt

**Keywords:** Valencene biosynthesis, S. guaranitica, Terpene synthase, Arabidopsis, Functional characterization, Protein domains and motifs

## Abstract

**Background:**

Salvia species produce a diverse array of terpenoids, particularly monoterpenes and sesquiterpenes, with applications in medicine, perfumery, and industry. However, the genes involved in sesquiterpene biosynthesis in *Salvia guaranitica* remain poorly characterized.

**Results:**

This study centers on the characterization of the *Valencene synthase SgTPS-V* from *S. guaranitica*. In silico analyses of *SgTPS-V* revealed conserved domains and motifs characteristic of terpene synthases, including DDxxxD, RWW, RxR, and NSE/DTE, and the phylogenetic tree placed *it* within the TPS-a subfamily, which encodes mono- and sesquiterpene synthases. Overexpression of *SgTPS-V* in *Arabidopsis thaliana* resulted in enhanced flowering relative to the wild-type. Also, comparing the chemical profiles of the transgenic Arabidopsis plants to wild-types indicated that *SgTPS-V* catalyzes the synthesis of Valencene, (-)-Valencene, (-)-Ledol, Viridiflorine and Valencene (isomer I), suggesting its role in sesquiterpene biosynthesis. Moreover, in vitro enzyme assay analysis for recombinant SgTPS-V protein showed that SgTPS-V had the capacity to convert FPP to considerable amount of Valencene and other amounts of sesquiterpenes. And based on results the major product for SgTPS-V was designated as a Valencene synthase.

**Conclusions:**

These results provide light on the molecular basis of sesquiterpene production in *S. guaranitica* and pave the way for the discovery of other terpene synthase genes in this species.

**Supplementary Information:**

The online version contains supplementary material available at 10.1186/s12870-026-08095-8.

## Highlights

Molecular cloning and characterization of Valencene synthase gene from *S. guaranitica*.

Putative tissue expression pattern of *SgTPS-V* gene under different tissues.

Putative conserved domains and motifs characteristic of *SgTPS-V* gene.

**Supplementary Information:**

The online version contains supplementary material available at 10.1186/s12870-026-08095-8.

## Background

*Salvia guaranitica* L., an Asian plant, belongs to Lamiaceae family, and is distributed across various provinces in China [1]. Its tissues exhibit high concentrations volatile (essential) oils, including numerous terpene derivatives such as α-Pinene, Cis-α-terpineol, (-)-Camphor, β-Caryophyllene, laevo-β-Pinene, Germacrene-A, Thujone, Phytan, Squalene, Farnesan, Ledol, 1,8-cineol, and (E) Phytol [1, 2]. These compounds are beneficial for their anti-inflammatory, antibacterial, high-grade lubricant, antitumor, antiseptic, spasmolytic, antioxidant, analgesic, cardiovascular, anti-cholinesterase, and antidiabetic properties [1, 3–10].

Terpenes represent a significant category of secondary plant metabolites, encompassing over 60,000 distinct structures [11]. All of these compounds are derived from small five-carbon units via either the mevalonic acid (MVA) pathway or the methyl-D-erythritol phosphate (MEP) pathway. Subsequently, these block units are linearly coupled to generate geranyl diphosphate (GPP) and farnesyl diphosphate (FPP) through cyclization, rearrangement, besides various modifications [11, 12]. Diverse structures, including monoterpenes (C10H16), sesquiterpenes (C15H24), diterpenes (C20H32), sesterterpenes (C25H40), triterpenes (C30H48), sesquarterpenes (C35H56), and tetraterpenes (C40H64), were synthesized by various terpene synthase (TPS) enzymes utilizing geranyl diphosphate (GDP), farnesyl diphosphate (FDP), and geranylgeranyl diphosphate (GGDP) as substrates [9, 13–17]. Furthermore, numerous full and partial cDNAs for mono- (C10), sesqui- (C15), di- (C20), sester- (C25), tri- (C30), and tetraterpene synthase genes (C40) have been identified, cloned, and characterized from the Lamiaceae family, particularly within the genus Salvia [17–22]. The previously studied terpene synthase enzymes exhibit various putative motifs in their C-terminal and N-terminal domains, which are crucial for determining product specificity [1, 14, 20, 23–29].

In recent years, NGS-based RNA sequencing (RNA-Seq) has become an effective tool for providing information about the genes which are related with secondary metabolite pathways in various medicinal plants, especially plants that belong to the lamiaceae family [1, 20]. The information gained from RNA-Seq studies help researchers for identification and discovery of novel genes and their Key Enzymes [1, 20, 30]. Furthermore, the purpose of identifying these genes is not only to facilitate functional studies but also to develop biotechnology for improving the production of medicinal ingredients, plant growth and defense through metabolic engineering [1, 2, 9, 10, 30, 31]. Through our numerous previous studies, we have successfully identified, characterized, and quantified the expression levels of several genes which related to terpene synthesis in various plants (e.g., *S. guaranitica*, *Salvia officinalis* and *Glycine max*), based on the data obtained from RNA-Seq data analyses such as; *SgCINS*,* SgGPPS*,* SgFPPS*,* SgCMS*,* SgTPS-V*,* SgHUMS*,* SgNEOD-1*,*SgNEOD-2*,*SgNEOD-3*,*SgTPS-1*,*SgTPS-3*,*SgTPS-6*,,* SgTPS4*,* SgLINS-1*,*SgLINS-2*,* SgGLNS*,* SgGERIS*,* SgFARD*,* SoAMYS*,* SoFLDH*,* SoTPS3*,* SoTPS6*,* SoNEOD*,* SoLINS*,* SoSABS*,* SoGPS*,* SoCINS*,* GmFDPS* and *GmGGPPS* [1, 2, 9, 10, 30, 31].

Functional analysis of mono- and sesquiterpene synthase genes indicates that each terpene synthase gene typically produces specific substantial products. However, some of these genes possess the capability to synthesize varying amounts of products through deprotonation or cyclization of carbocations [1, 17, 20, 25–31]. Prior investigations elucidated the potential role of Salvia terpene synthases in terpene biosynthesis [1, 2, 17, 20, 29–32]. The article focus on the functional characterization of *SgTPS-V*, identified as the Valencene synthase gene, elucidating its biochemical role in sesquiterpene synthases in *S. guaranitica*.

## Materials and methods

### In silico Studies

The *SgTPS-V* was chosen as an authentic ortholog to other plant sesquiterpene syntheses [1]. Using the Arabidopsis transcript expression database, expression data for *S. guaranitica* sixty tissues were collected. The profiles of the transcript expression in different plant organs were created via eFP Browsers from Arabidopsis (eFP Browsers (http://bar.utoronto.ca/efp/cgi-bin/efpWeb.cgi). The profile expression pointers (arrows) show the degree of expression - red means high expression and yellow means lesser expression). Additionally, putative localizations of *SgTPS-V* from *S. guaranitica* was verified on the basis of Arabidopsis protein localization patterns at fourteen cell organelles to identify possible synthesis sites with Cell-eFP (http://bar.utoronto.ca/cell_efp/cgi-bin/cell_efp.cgi) eFP Browsers. Also, PROTPARAM Server (http://web.expasy.org/protparam/) elucidated the physiochemical characters of SgTPS-V. Moreover, iPSORT (http://ipsort.hgc.jp/), WolF PSORT Prediction (https://www.genscript.com/wolf-psort.html), TargetP-2.0 (https://services.healthtech.dtu.dk/services/TargetP-2.0/) and DeepLoc − 2.0 (https://services.healthtech.dtu.dk/services/DeepLoc-2.0/) were used to highlight SgTPS-V signal peptide based on the highest similarity scores with other plant signaling proteins. SgTPS-V was analyzed comparatively using the NCBI’s BLASTX to identify authentic homologs proteins (http://blast.ncbi.nlm.nih.gov/). For homology-based comparison, the online Clustal Omega was employed (https://www.ebi.ac.uk/Tools/msa/clustalo/). Also, a maximum-likelihood phylogenetic tree was constructed using the PhyML version 3.0 Server, with default settings and 1000-repetition bootstrapping to scrutinize the evolution of history of SgTPS-V with other orthologous plant TPS genes [32, 33]. And the protein sequences of functionally characterized TPS genes were retrieved from the National Center for Biotechnology Information (NCBI) database.

### Plant material, cDNA library and cloning of the full-length region of cDNA

Complementary DNA (cDNA) was provided from our previous work on *S. guaranitica* [9], and the plant material was collected previously from the Wuhan Botanical Garden, Wuhan, China (Geo URI: geo:30.54445,114.42329). Concisely, TransZol Reagent was used to extract total RNA while cDNA was synthesized using the Super Mix kit ([1, 20, 34]. For gene cloning, the full-length cDNAs of *SgTPS-V* (GenBank: KX893974.1) was retrieved from NCBI for primer designing [1]. A pair of short and long degenerative primers was designed as short forward: 5′-ATGAGTGTTTTATTGTCAACAACTACT C-3′, short reverse: 5′-TTACGTTCTA TTATTGACACAAATAAAATA-3′, long forward: 5′-GGGGACAAGTTTGTACAAAAAAGCAGGCTTCATGAGTGTTTTAT TGTCA − 3′ and long reverse: 5′-GGGGACCACTTTGTACAAGAAAGCTGGGTTT ACGTTCTATTATTGAC-3′. Short primers and cDNA were used for the first PCR, after which the resultant PCR product was used along with long gene-specific primers. The final amplified fragments were then introduced in the pDONR221 vector (Invitrogen, Carlsbad, CA, USA) after purification [1, 20, 34]. Subsequently, the amplified *SgTPS-V* cDNA was transferred to the pB2GW7 vector (Invitrogen, Carlsbad, CA, USA). To authenticate the successful insertion, sequencing was performed using vector specific forward with *SgTPS-V* reverse primers for positive colonies selection, the recombinant vector preparation, transferred into bacteria, sequencing and cloning system were performed [1, 9, 20, 29–32].

### Preparation of cDNA library from purified RNA

For Semi-quantitative RT-PCR and qRT-PCR, the total RNA from the leaves of wild type and transgenic *A. thaliana* were collected using Transzol Reagant (Focus Bioscience, Australia). The quantification and purity of the extracted RNA was assessed with NanoDrop™ 2000/2000c Spectrophotometers (Nanodrop technologies, Wilmington, DE, USA), and visualized on a 1.64% (W/V) agarose gel. Then, 1 µg RNA was used to synthesize cDNA via TransScript^®^ First-Strand cDNA Synthesis Super Mix Kit (TransGen Biotech Beijing, China) as of the manufacturer’s instructions [20, 34].

### Genetic transformation

*Arabidopsis thaliana* ecotype Columbia-0 (Col-0) were cultivated according to Ali et al., 2018 and Mohammed et al., 2023a [1, 31]. Healthy pre-flower plants were selected for the floral-dip transformation experiment two months after growth. *Agrobacterium tumefaciens* strain GV101 received pB2GW7-SgTPS-V (expression vector) and pB2GW7 (empty vector). *A. tumefaciens* containing the expression vector was grown on Rif and Spc-supplemented solid LB medium. After screening, a positive colony was injected into 0.8 mL of liquid LB medium with the same antibiotics and components. Briefly, as stated by Ali et al. 2017; 2018 and Darwish et al. 2022) [1, 20, 35, 36], Agrobacterium cells were sub-cultured in Luria-Bertani medium or Lysogeny broth (LB medium) and incubated in a shaker incubator till they growth of 0.76 at the optical density at 600 nm (OD600). Then, a floral-dip inoculation medium with 5.2% sucrose and 0.055% Silwet was used to resuspend the pellet from Agrobacterium cell suspension. *A. thaliana* was individually introduced by immersing secondary inflorescence explants in fresh floral dip inoculation medium and applying gentle pressure to facilitate the uptake of *A. tumefaciens* vectors into the flowers’ gynoecia. A sequential culture regimen utilizing BASTA selection was applied to select the suitable plants from T0 and T1 and finally we successfully selected 12 transgenic *A. thaliana* transgenic lines from T2. Transgenic lines were investigated for leaf morphology, flowering time, and terpene metabolism.

### Gene expression analysis

In verification of achieved gene transfer results, Semi-Quantitative RT-PCR (sqRT-PCR) was applied using an Eppendorf PCR system (Mastercycler Nexus PCR Machine from Eppendorf, Sydney, Australia). The housekeeping *At-B-actin* forward primer was 5’-GGCTGAGGCTGATGATATTC-3’ and reverse primer was 5’-CCTTCTGGTTCATCCBAAC-3’ to amplify 155 bp. However, *SgTPS-V* forward primer was 5’-GTTACTTCTGGGCGTTGG-3’ and the reverse one was 5’- CATCATCCCACCTCTGTA TTG − 3’ to amplify 168 bp. The PCR conditions were: 96 °C for 2 min, 94 °C for 35 s, 58.5 °C for 40 s, 72 °C for 30 Sect. (35 cycles), then a final extension at 72 °C for 10 min. Also, agarose gel was extra used to validate *SgTPS-V* expression analysis [37–40]. To analyze the expression of *SgTPS-V* at transgenic *A. thaliana* and wild type, qRT-PCR was performed using IQTM5 System, SYBR Green, and the cycler program as follows: 94 °C for 11 s, 58.5 °C for 30 s, and 72 °C for 20 s, then 66 °C for 5 s and 94 °C for 6 s. Moreover, the same qRT-PCR primers and condations were used to validate the expression of *SgTPS-V* gene under different tissues from *S. guaranitica* plant (e.g, young leaf, old leaf, stem, flower, bud flower and root). The expression levels were enumerated by comparing our target gene cycle thresholds (CTs) with the housekeeping gene *SgACTIN* using the 2^-ΔΔCt^ method (20, 31, 39). Values were offered as means ± SE of three different RNA pool replicates.

### Terpenoids quantitative and qualitative analyses

GC-MS analysis was harnessed to compare terpenoids species in transgenic lines with wild type plants. For this, 12 leaves from each transgenic *A. thaliana* line or non-transgenic (three leaf from each plant) were homogenized in liquid nitrogen with a mortar and pestle, after which the plant material powder was directly soaked in n-hexane as a solvent in Amber storage bottles, 30 ml screw-top vials with silicone/PTFE septum lids (http://www.sigmaaldrich.com) were used to reduce loss of volatiles to the headspace then incubated with shaking at 37 °C and 200 rpm for 75 h. Afterward, the solvent was transferred using a glass pipette to a 10-ml glass centrifuge tube with screw-top vials with silicone/PTFE septum lids and centrifuged at 5,000 rpm for 10 min at 4 °C to remove plant debris [1,9, 20, 29, 30,]. A single microliter’s worth of the extract was put into a Shimadzu model GCMS-QP2010 Ultra and analyzed in triplicate. The Wiley-GC/MS-Library (10th Edition), the Volatile Organic Compounds (VOC) Analysis S/W software, and the NIST-Library (2014 edition) were used as references to identify various terpenoids [1, 2, 9, 20, 40–42].

### Expression of Valencene synthase (SgTPS-V) recombinant proteins in *Escherichia coli* and enzyme activity assay

The full-length sequences of *SgTPS-V* gene was transferred to the pDEST17 Gateway^®^ destination vector. To authenticate the successful insertion, sequencing was performed using vector specific forward with *SgTPS-V* reverse primers for positive colonies selection, the recombinant vector preparation, transferred into *Escherichia coli*)*E. coli(* strain BL21 (DE3) bacteria, sequencing and cloning system were performed [1, 9, 20, 29–32]. The recombinant proteins were induced under various concentration from isopropyl-β-d-thiogalactopyranoside (IPTG) (e.g., 0.1 mM, 0.2 mM, 0.3 mM and 0.4 mM) at 26 °C for 15 h. After induction, the bacteria cells were harvested by centrifugation under cooling, then the bacteria cell pellets will resuspended in phosphate-buffered saline, and disrupted by sonication machine. The crude proteins were then extraction using BugBuster^®^ Ni-NTA His•Bind^®^ Purification Kit (Milipore) according to the manufacturer’s instructions. The purified proteins were collected and concentrated before enzyme assays and they were examined by SDS–PAGE. as described previously [43–46].

The Valencene synthase activity assays were conducted as described [43–46] with some modifications. In briefly, the reaction mixture consist from 25 mM HEPES buffer (pH 7.4), 15 mM MgCl2, 5 mM dithiothreitol, 50 µg purified protein, 2 mM FPP (Sigma-Aldrich) as substrate, in a totally volume of 100 µl. The reaction mixtures were incubated at 30 °C for 2 h. After incubation, extraction of reaction products relied on n-hexane 250 µl, and resulting extract 2 µl was sent to GC-MS analysis. GC-MS conditions were consistent with that described a bove at terpenoids quantitative and qualitative analyses.

## Results

### Cloning and sequence analysis of the full-length *SgTPS-V*

The entire open reading frame of the *SgTPS-V*, comprising 1829 base pairs, encodes a protein of 604 aa, which is anticipated to have a theoretical IP of 5.35 and a molecular weight of 70.70 kDa. The hypothesized amino acid sequence of the *SgTPS-V* features a signal peptide that is shorter than those found in monoterpene synthases (600–650 aa) and sesquiterpene synthases (550–580 aa). Furthermore, our target protein sequence aligns with numerous sesquiterpene synthases due to the presence of a 30 amino acid long target sequence (MSVLL STTTQ PKFGI FRHIY TMSTV NSNSF) at the N-terminal. The application of the ‘iPSORT’ program, WolF PSORT Prediction, TargetP-2.0 and DeepLoc − 2.0 databases have elucidated that *SgTPS-V* protein have mainly localized in the Plastid, which maybe because the origin of FPP and its biosynthetic processes are in these organelles. The BLASTX analysis presented in Table 1 reveals that *SgTPS-V* exhibits a remarkable identity ranging from 91.45% to ≥ 66.60% with its homologous sesquiterpene synthase protein derived from *Salvia splendens* and various plant species.

**Table 1:** NCBI BLASTX results of *SgTPSV* to retrieve authentic homologous proteins

NCBI Accession	^a^ Description	Organism	E value	Identity (%)	Accession length
XP_042060897.1	(-)−5-epieremophilene synthase STPS3-like	*Salvia splendens*	0	91.45%	550
XP_042060899.1	(-)−5-epieremophilene synthase STPS3-like	*Salvia splendens*	0	84.39%	551
AAX16076.1	Putative sesquiterpene synthase	*Perilla frutescens* var. frutescens	0	69.56%	550
AGN72800.1	Germacrene A	*Lavandula pedunculata* subsp. lusitanica	0	70.93%	547
XP_022850114.1	Vetispiradiene synthase 2-like	*Olea europaea* var. sylvestris	0	68.27%	554
QIQ55988.1	Putative terpene synthase 2	*Eremophila denticulata* subsp. trisulcata	0	68.06%	557
XP_011073749.1	Vetispiradiene synthase 3	*Sesamum indicum*	0	67.70%	554
QNC49793.1	Terpene synthase 12a	*Leucophyllum frutescens*	0	66.61%	553
A0A1W6GW18.1	RecName: (-)−5-epieremophilene synthase	*Salvia miltiorrhiza*	0	68.01%	546
A0A1W6GW06.1	RecName: (-)−5-epieremophilene synthase	*Salvia miltiorrhiza*	0	68.01%	546
AGN72803.1	Germacrene A	*Lavandula stoechas*	0	69.39%	542
AAX16077.1	Valencene synthase	Perilla frutescens var. frutescens	0	67.22%	550
A0A1W6GW32.1	RecName: (-)−5-epieremophilene synthase	*Salvia miltiorrhiza*	0	67.65%	546
AGN72806.1	Germacrene A	*Lavandula viridis*	0	69.26%	543
CAA3024998.1	Vetispiradiene synthase 2-like	*Olea europaea* subsp. europaea	0	66.67%	555
GER46991.1	Valencene synthase	*Striga asiatica*	0	66.60%	556

^a^Description—homology search using BLASTX

**Fig. 1:**
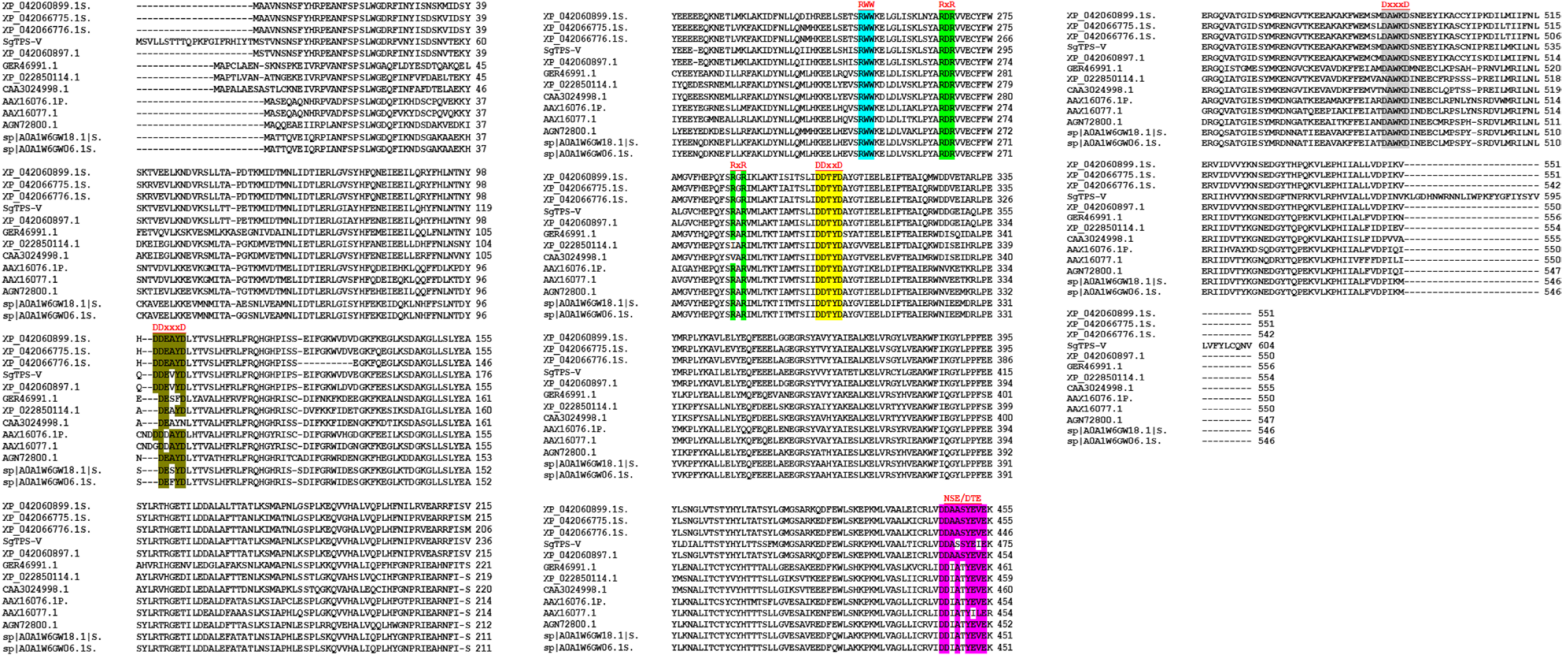
Multiple sequence alignment. The deduced amino acid sequence of SgTPS-V was aligned with homologues identified from the BLASTX analysis. The conserved motifs DDEVYD, RWW, RxR, DDxxD, NSE/DTE and DxxxD are marked. SgTPS-V: Valencene synthase from *S. guaranitica *plant

The alignment of SgTPS-V with other authentic orthologous sequences facilitated the inference of its potential function. According to this prediction, the SgTPS-V exhibits a variety of motifs, including DDEVYD (residues 121–126), RWW (residues 271–273), RxR (residues 286–288), RxR (residues 307–309), DDxxD (residues 323–327), NSE/DTE (residues 466–474), and DxxxD (residues 508–512). These motifs are prevalent in analogous sesquiterpene synthases associated with Valencene synthase [1, 9, 20, 27, 29, 47, 48] (Fig. 1).

**Fig. 2:**
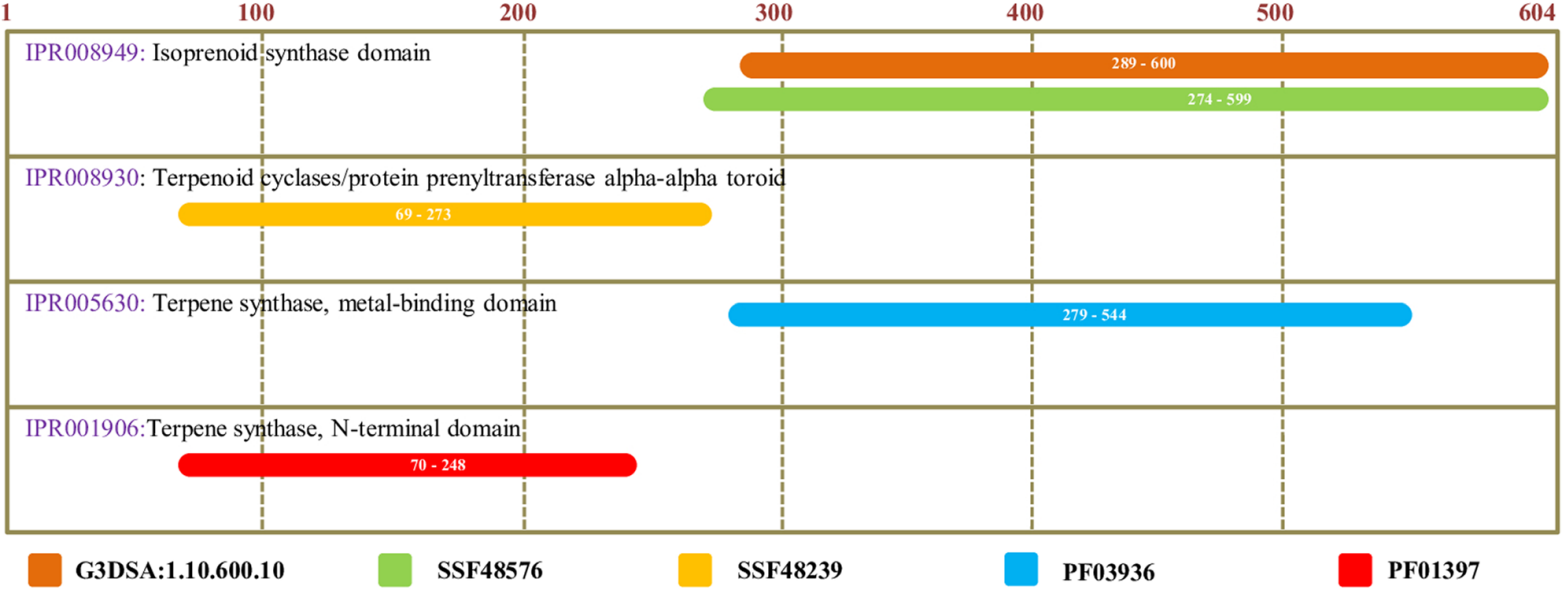
Putative domain analysis for SgTPS-V using the InterPro protein sequence analysis & classification (https://www.ebi.ac.uk/interpro/) database. SgTPS-V protein sequence has four protein family domains

**Fig. 3:**
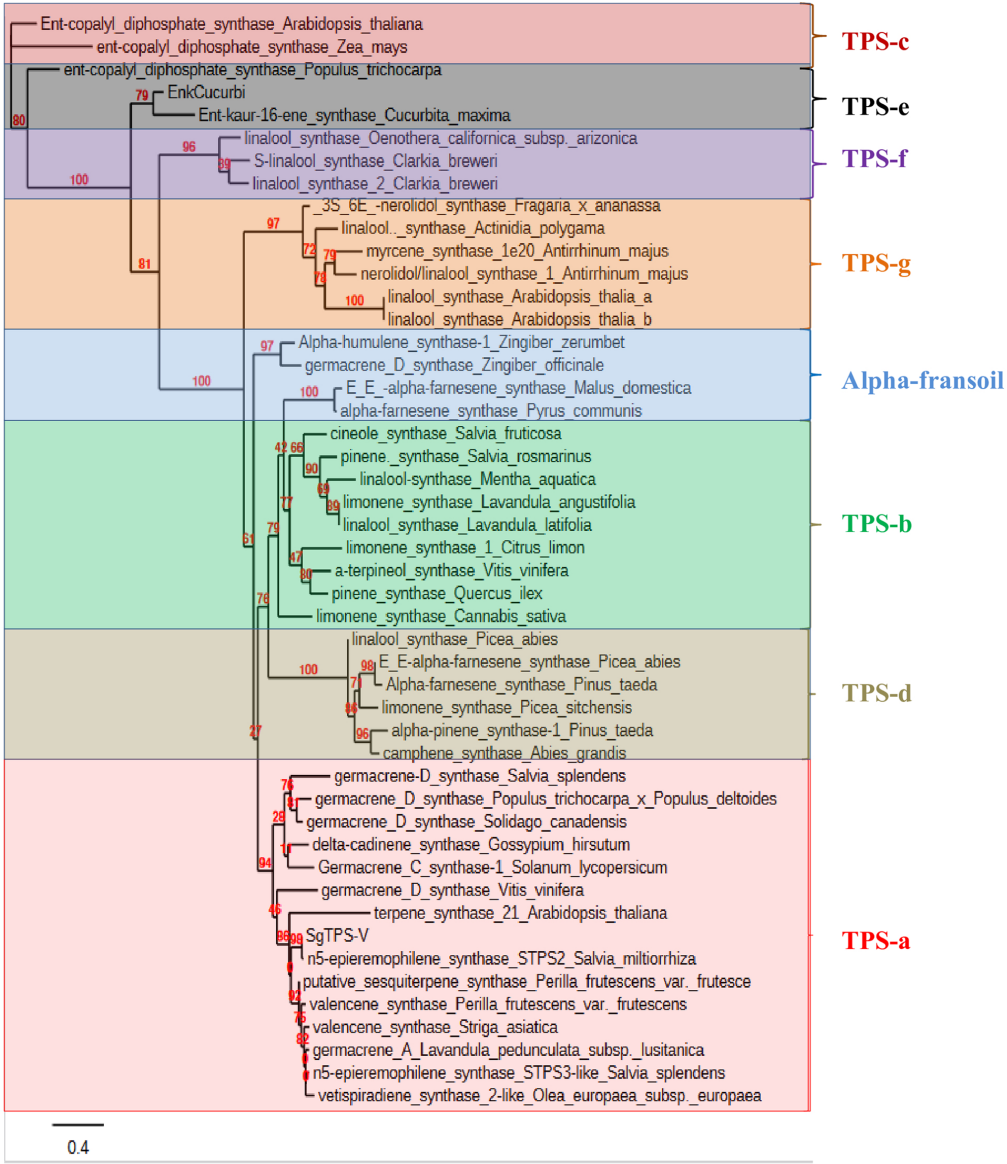
Phylogenetic tree of SgTPS-V with selected terpene synthases from other plants. Seven previously identified TPS subfamilies (Tps-a to Tps-g) were chosen based on Degenhardt et al., 2009; Abdelhameed et al., 2024; Esraa et al., 2022; Nasir et al., 2021 [49–52]. The alignment was performed using the PhyML server. The numbers indicated are the actual bootstrap values of the branches

In comparison to other sesquiterpene synthases, SgTPS-V is characterized by five distinct domains, as delineated by the InterPro protein sequence analysis and classification database (InterPro: https://www.ebi.ac.uk/interpro/). The SgTPS-V protein comprises five distinct domains belonging to the terpene synthase family: the Terpenoid cyclases/protein prenyltransferase alpha-alpha toroid (IPR008930: spanning residues 69 to 273), the Terpene synthase N-terminal domain (IPR001906: spanning residues 70 to 248), the Terpene synthase metal-binding domain (IPR005630: spanning residues 279 to 544), the Farnesyl Diphosphate Synthase (1.10.600.10: spanning residues 289 to 600), and the Isoprenoid synthase domain (IPR008949: spanning residues 274 to 599) (see Fig. 2). Furthermore, the SgTPS-V has been categorized within the TPS-a subfamily of angiosperm sesquiterpene synthases, as indicated by the results of the phylogenetic analysis (Fig. 3).

### In Silico expression profiling and subcellular localization of *SgTPS-V*

**Fig. 4:**
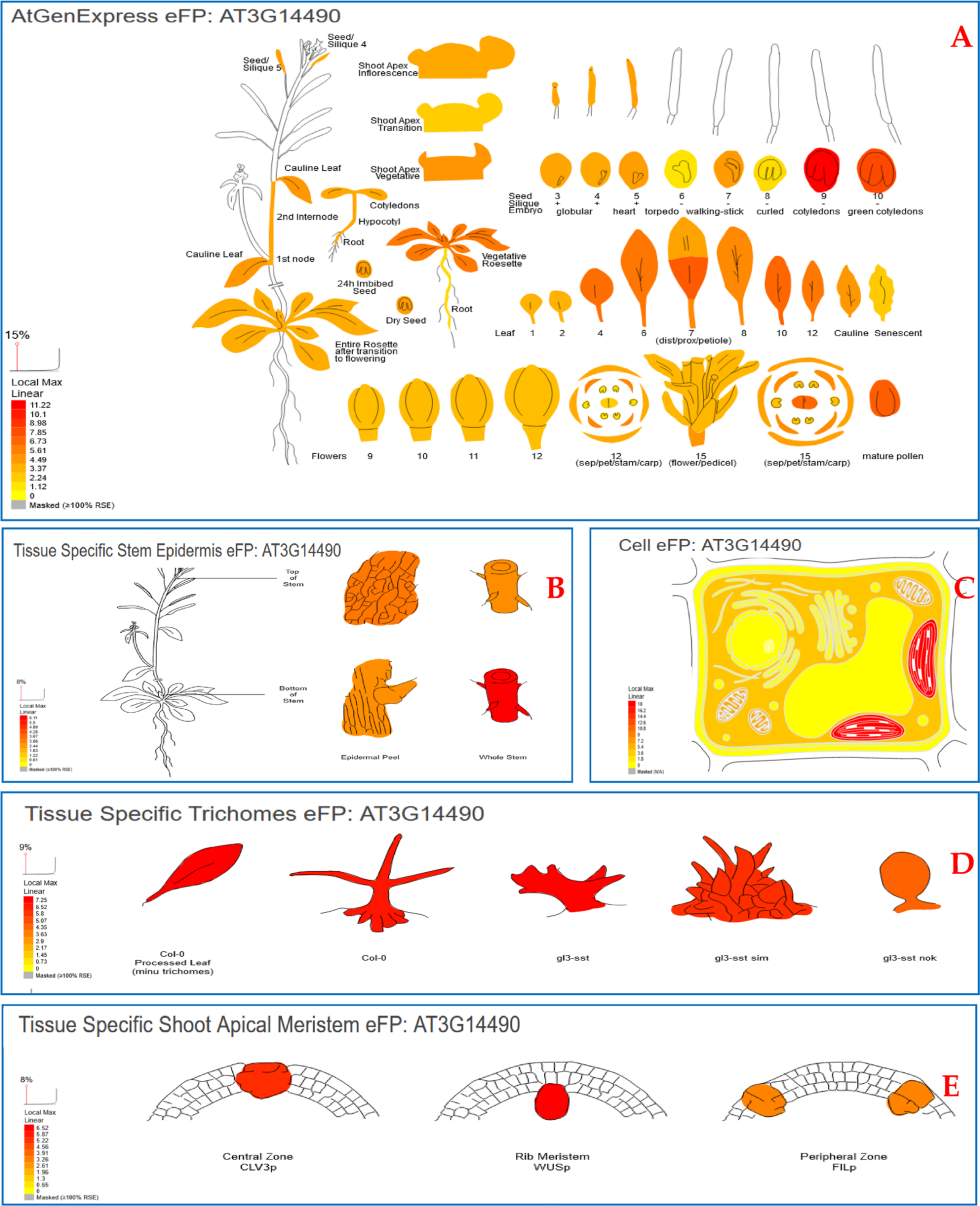
Visualization the putative an “electronic fluorescent pictograph” browsers for exploring the putative tissue expression and cell localization of SgTPS-V (AT3G14490) gene, based on Arabidopsis gene expression and protein localization at different tissues and cell organs. **a** Expression data at different tissues from seedling to flowering stages. **b** Expression data of tissue specific stem epidermis at top and bottom. **c** Expression data at different cell organs. **d** Expression data tissue at specific trichomes. **e** Tissue Specific Shoot Apical Meristem. The color box represents the expression scale (the more intense red color the more gene expression)

A BlastP search was conducted using the SgTPS-V protein sequence as a query to investigate the hypothesized tissue expression pattern of SgTPS-V in the *A. thaliana* genome at the Phytozome database (https://phytozome.jgi.doe.gov/pz/portal.html#!search? show=BLAST&method=Org_Athaliana). This study found other proteins closely associated with the SgTPS-V sequences, particularly (AT3G14490), which had a high BLAST score and e-value of 351 and 1.62e-113, respectively. The hypothesized tissue expression patterns of the *SgTPS-V* in Arabidopsis, revealed by our data, were analyzed across forty-five tissues using the BAR database (http://bar.utoronto.ca/efp/cgi-bin/efpWeb.cgi) (Fig. 4A). The Arabidopsis eFP Browsers indicated that *SgTPS-V* (AT3G14490) was predominantly expressed in various tissues, particularly in Seeds Stage 9 without Siliques (11.22), Seeds Stage 10 without Siliques (8.03), Leaf 7, Proximal Half (7.28), Mature Pollen (7.07), Flower Stage 15, Carpels (6.73), Rosette Leaf 12 (6.72), and Rosette Leaf 4 (6.37) (Fig. 4a and Supplementary Table S1). Conversely, this gene exhibited significant expression in tissue-specific stem epidermis, particularly at the apex of the stem (3.28), followed by the entire stem, apex of the stem (2.72), stem epidermis, base of the stem (2.69), and the entire stem, base of the stem (6.11) (Fig. 4B and Supplementary Table S1).

Furthermore, Arabidopsis Cell Electronic Fluorescent Pictograph tool (eFP; http://bar.utoronto.ca/cell_efp/cgi-bin/cell_efp.cgi) was used to predict the putative subcellular localizations of our SgTPS-V (AT3G14490) gene according to the protein localization of different cell organelles in the Arabidopsis. The subcellular localization profiles showed that SgTPS-V protein was highly expressed and presented in the Plastid (see Fig. 4C). Also, it was present with highly expressed in all tissue specific trichomes (Fig. 4D and Supplementary Table S1). Ultimately, our target gene exhibited high expression in the tissue-specific shoot apical meristem, particularly in the Central Zone (5.38), followed by the Rib Meristem (6.52) and the Peripheral Zone (3.16) (Fig. 4E and Supplementary Table S1).

### Characterization of *SgTPS-V *Activity in transgenic *Arabidopsis thaliana *leaves and validation of the *SgTPS-V* gene expression patterns by quantitative RT-PCR

**Fig. 5:**
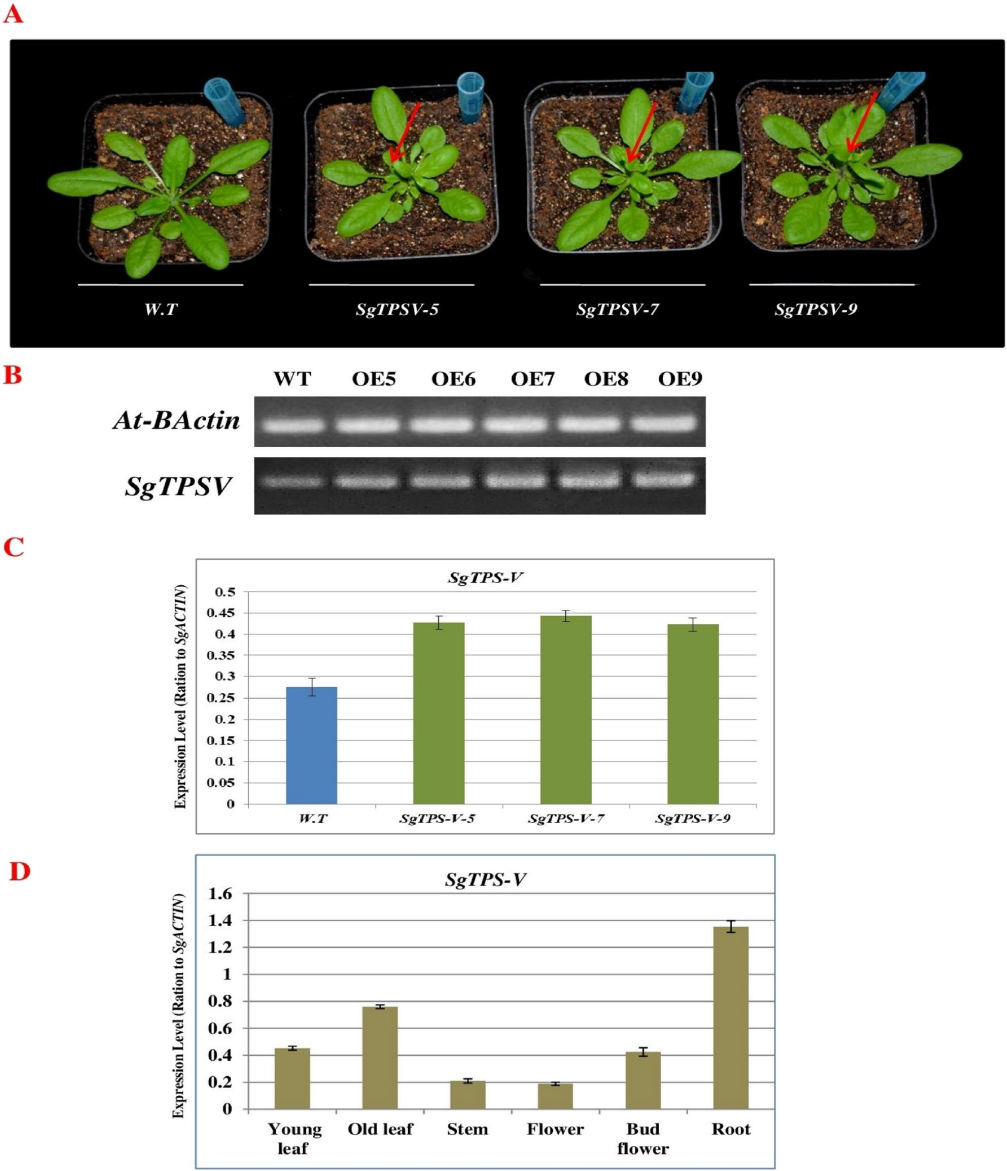
Overexpression of *SgTPS-V* gene in transgenic Arabidopsis. **A** Comparison of the phenotypes of the transgenic *A. thaliana* and wild type A. thaliana. The red arrow (↘) indicates the flowers at the transgenic A. *thaliana*. **B** Semiquantitative RT-PCR to confirm the expression of terpenoid genes. **C** Quantitative RT-PCR validation of expression of *SgTPS-V* gene at transgenic *A. thaliana* and wild type. **D** Quantitative RT-PCR validation of expression of *SgTPS-V* gene under different tissues of *S. guaranitica* Plant

The *SgTPS-V* was cloned from *S. guaranitica* to examine its impact on the phenotypes of *A. thaliana* plants after 36 days of growth. Subsequently, *A. thaliana* was employed as a transient bio-expression system to overexpress *SgTPS-V*. We employed the *A. tumefaciens*-GV101 containing the vector pB2GW7-SgTPS-V, regulated by the 35 S-promoter, to produce transgenic *A. thaliana* plants that constitutively overexpress the *SgTPS-V* (Fig. 5A). The positive transforming lines were then validated using both BASTA reagent and semi-quantitative RT-PCR of the cDNA (Fig. 5B). QRT-PCR was used to inspect the transcription levels of *SgTPS-V* at transgenic *A. thaliana* and wild type. (Fig. 5C). From the qRT-PCR analysis results, it was found that the highest expression levels were observed in various transgenic *A. thaliana* lines in compared with the wild type (Fig. 5C). Moreover, qRT-PCR was used to validate the expression patterns of SgTPS-V gene across different tissues of *S. guaranitica* such as; young leaf, old leaf, stem, flower, bud flower and root, to understand their expression profiles within the previous various tissues samples (Fig. 5D). Transgenic *A. thaliana* plants exhibited an increase in leaf width and an earlier onset of flowering stem growth compared to the wild type plants.

### Profiling terpene contents

**Fig. 6:**
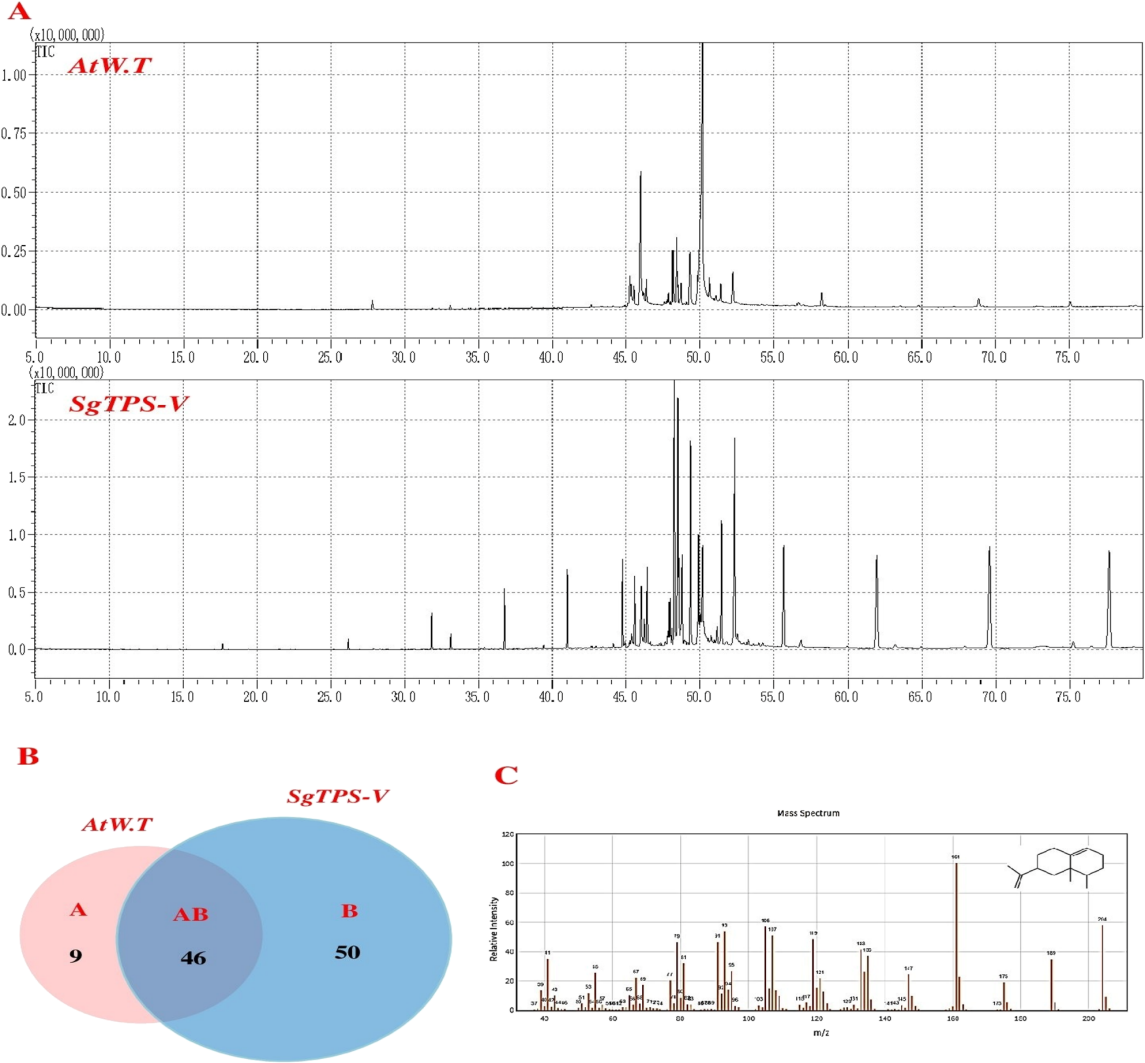
Typical GC-MS mass spectrographs for terpenoids from leaf of *A. thaliana* plants. **A** GC-MS Peak of the essential oil, (**B**) Two-way Venn diagram to show the number of unique and common compounds in the essential oil extracts from the transgenic and non-transgenic *A. thaliana*. (C) The Mass spectrum (electron ionization) and structure of valenene

To investigate the impact of *SgTPS-V* overexpression in the leaves of *A. thaliana*, the qualitative and quantitative alterations in terpene profiles were monitored through GC-MS analysis. The findings indicated that multiple terpenes exhibited a significant increase in transgenic *A. thaliana* leaves with *SgTPS-V* overexpression, in comparison to the control, as detailed in Table 2 and illustrated in Fig. 6A. In leaves of *A. thaliana* plants upregulating *SgTPS-V*, sesquiterpene compounds constituted the predominant group at 48.08%, followed by diterpene compounds at 8.42% and monoterpene compounds at 0.51%. Additionally, one triterpene compound was represented at 0.15%. In non-transgenic *A. thaliana* (control), sesquiterpene compounds constituted the predominant group at 14.85%, followed by diterpene compounds at 10.26% and monoterpene compounds at 0.1%, see (Table 2). The hexane extracts from transgenic and non-transgenic *A. thaliana* exhibit distinct, shared, and predominant compounds (Table 2 and Fig. 6B).

**Table 2:** Principal terpenoids detected in Transgenic *A. thaliana* leaves that overexpress *SgTPS-V*

*N*	Compounds	*R*.T (min.)	Formula	Molecular Mass (g mol-1)	Type	% Peak area	
						AtW.T	SgTPS-V
1	(8)Annulene	5.963	C8H8	104.1491		0.06	0.03
2	Undecane	17.649	C11H24	156.308		0.01	0.21
3	α-Terpinenyl acetate	26.194	C12H20O2	196.286	Mono	0.01	0.4
4	Patchulane	31.833	C15H26	206.367	Sesqui	0.04	1.35
5	Trans-β-Ionone	32.304	C13H20O	192.2973		0.06	0.03
6	Topanol	33.074	C15H24O	220.3505	Sesqui	0.18	0.58
7	All-trans-Geranylgeraniol	35.132	C20H34O	290.4834	Diter		0.03
8	p-Mentha-6,8-dien-2-ol, cis	37.027	C10H16O	152.2334	Mono	0.09	
9	Isovaleric acid p-tolyl ester	37.369	C12H16O2	192.2542	Mono		0.01
10	Guaia-1(10),11-diene	44.898	C15H24	204.3511	Sesqui	0.09	0.13
11	β-cedren-9-α-ol	45.113	C15H24O	220.3505	Sesqui	0.28	0.13
12	Cedrenol	47.234	C15H24O	220.3505	Sesqui		0.18
13	β-Carotene	47.66	C40H56	536.8726	Triter		0.15
14	Ledol	47.774	C15H26O	222.3663	Sesqui	0.24	0.55
15	(+)-Valencene	47.873	C15H24	204.3511	Sesqui	0.66	1.78
16	Kolavenol acetate	47.989	C22H36O2	332.52	Diter	0.15	0.63
17	Geranylgeraniol	48.136	C22H36O2	332.52	Diter	3.88	
18	Valencene	48.394	C15H24	204.3511	Sesqui	4.09	13.24
19	(-)-Valencene	48.464	C15H24	204.3511	Sesqui	1.16	9.13
20	Valencene (isomer I)	48.692	C15H24	204.3511	Sesqui	1.87	3.25
21	4,8,13-Duvatriene-1,3-Diol	49.272	C20H34O2	306.4828	Diter	2.63	
22	Phytol	49.319	C20H40O	296.531	Diter	3.6	7.6
23	Viridiflorine	49.825	C15H24	204.3511	Sesqui	2.11	4.04
24	Linolenic acid	50.164	C18H30O2	278.4296		44.78	1.13
25	4,8,13-Duvatriene-1,3-Diol	50.5	C20H34O2	306.4828	Diter		0.16
26	(+)-Ledol	51.048	C15H26O	222.3663	Sesqui	0.41	0.66
27	(-)-Ledol	52.208	C15H26O	222.3663	Sesqui	2.95	7.83
28	Epiglobulol	52.523	C15H26O	222.3663	Sesqui		0.42
29	Ledol, isomer 1	52.677	C15H26O	222.3663	Sesqui		0.12
30	trans-caryophyllene oxide	53.221	C15H24O	220.3505	Sesqui		0.2
31	α-Elemol	53.954	C15H26O	222.3663	Sesquit		0.1
32	Elemol	54.147	C15H26O	222.3663	Sesqui	0.09	0.12
33	Z-Citronellyl tiglate	55.724	C15H26O2	238.3657	Sesqui	0.09	3.8
34	Ledane	56.633	C15H26	206.3669	Sesqui	0.29	0.03
35	β-Elemol	56.961	C15H26O	222.3663	Sesqui	0.3	0.26
36	Isovaleric anhydride	73.242	C10H18O3	186.2481	Mono		0.08
37	(-)-Myrtenol	78.184	C10H16O	152.2334	Mono		0.02
	Total % of monoterpene					0.1	0.51
	Total % of sesquiterpene					14.85	48.08
	Total % of diterpene					10.26	8.42
	Total % of triterpene						0.15

### Measurements of enzyme activity

**Fig. 7:**
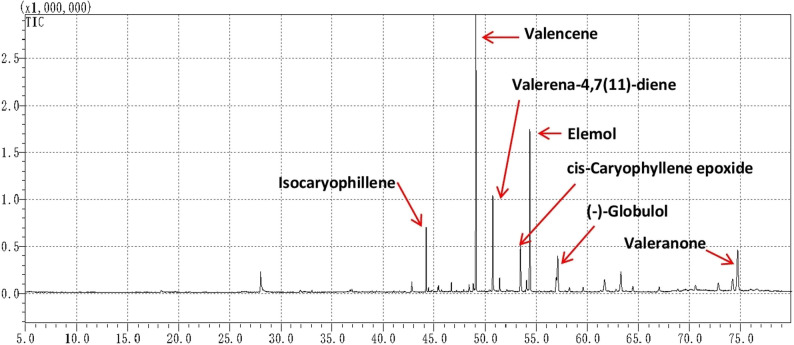
GC-MS of the products generated in vitro by recombinant SgTPS-V protein. In vitro enzymatic activity analysis of SgTPS-V protein using FPP as substrate Products catalyzed by SgTPS-V protein using FPP as substrate were subjected to GC-MS. Peaks were identified as terpenes by comparing mass spectra with various library such as Wiley-GC/MS-Library (10th Edition), the Volatile Organic Compounds (VOC) Analysis S/W software, and the NIST-Library (2014 edition), that used as references to identify various terpenoids

To express SgTPS-V proteins in *E. coli*, the full-length sequences of *SgTPS-V* genes were amplified with Gateway method and then sub cloned into the pDEST17 Gateway^®^ destination vector. Then the recombinant proteins induced various concentrations from IPTG and from our results we found the optimal induction concentration and condition was 0.4 mM at 26 °C for 15 h. Furthermore, the crude proteins were then extraction using BugBuster^®^ Ni-NTA His•Bind^®^ Purification Kit (Milipore) and the purified proteins were isolated and electrophoresed by SDS–PAGE see Supplementary Fig. S1. Moreover, enzymatic assays of the crude SgTPS-V *E. coli* extracts showed that a fully functional protein was being produced and the reaction products were extracted in 250 µl of hexane and the reaction products were running using GC-MS for analysis. And from GC-MS analysis we found the major compound produce by SgTPS-V enzyme in vitro assay is Valencene (38.01%) at 48.923RT, followed by Elemol (12.06%) at 54.147 RT, Valerena-4,7(11)-diene (9.47%) at 50.65 RT, Isocaryophillene (7.12%) at 44.15 RT, cis-Caryophyllene epoxide (5.1%) at 53.221 RT, Valeranone (4.35%) at 74.53RT, and (-)-Globulol (3.86%) at 57.025 RT (see Fig. 7).

## Discussion

The complete cDNA of the SgTPS-V was identified and extracted from the pool of leaves of *S. guaranitica*, utilizing in silico sequence comparison with other orthologs from *S. splendens*,* Lavandula pedunculata* subsp. lusitanica, *Salvia miltiorrhiza*, *L. stoechas*, and *L. viridis*. In comparison to other sesquiterpene synthases, the SgTPS-V contains five domains, as identified by the InterPro protein sequence analysis and classification database (InterPro: https://www.ebi.ac.uk/interpro/). The first domain is the Terpenoid cyclases/-protein prenyltransferase alpha-alpha-toroid (IPR008930: residues 69–273), followed by the Terpene synthase, N-terminal domain (IPR001906: residues 70–248). The third to fifth domains include the Terpene synthase, metal-binding domain (IPR005630: residues 279–544), Farnesyl Diphosphate Synthase (1.10.600.10: residues 289–600), and the Isoprenoid synthase domain (IPR008949: residues 274–599) (Fig. 2). A comparison of the putative SgTPS-V with other orthologous proteins revealed several highly conserved motifs, including DDxxD (residues 323–327) and DxxxD (residues 508–512). These conserved motifs are located close to the active site. Therefore, they may stabilize the binding of the substrate and the inorganic cofactor [1, 20, 27, 48, 49, 53, 54] (Fig. 1).

Additionally, these conserved motifs stabilize a tri-nuclear magnesium cluster [27, 53, 55], which in turn catalyzes the formation of the C15-substrate-FPP at the hydrophobic substrate-binding-pocket [27, 49, 56]. Additionally, RWW (residues 271–273), RxR (residues 286–288), and RxR (residues 307–309) are essential for product cyclization in class-III-TPS proteins [48, 57, 58] (Fi. 1). The in-silico studies identified also additional conserved region motifs in the SgTPS-V, specifically the DDEVYD (residues 121–126) and NSE/DTE (residues 466–474) regions, which are prevalent in other sesquiterpene synthases. Each protein sequence within the terpene synthase family contains one or two conserved domains and motifs [1, 17, 20]. The phylogram was constructed to examine the phylogenetic relationships among SgTPS-V and other sesquiterpene synthases, utilizing the neighbor-joining method for optimal parameter selection. The results indicate that SgTPS-V belongs to the TPS-a subfamily, which is capable of encoding sesquiterpenes synthase. This result supports the previously noted ability of SgTPS-V to produce various types of terpenes [1, 20] (Fig. 3).

The in-silico expression patterns of SgTPS-V across forty-five tissues were investigated harnessing the high similarity between SgTPS-V and its homologs sequence from *A. thaliana* (AT3G14490). The SgTPS-V in-silico expression patterns were consistent with findings from Ali et al. (2017, 2018, 2022a) [1, 9, 20], which indicated that the majority of TPS genes (e.g., *SoFLDH*,* SgTPSV*,* SgGERIS*,* SoLINS-2*,* GmTPS-21*,* SgFARD*,* SoNEOD*, and *SoHUMS*) from *S. officinalis*, *Glycine max*, and *S. guaranitica* exhibited high transcripts in seeds, leaves, mature pollen, stems, trichomes, and flowers. The proposed subcellular localization of the SgTPS-V protein indicates that it is primarily found in the plastids, mitochondria, and cytosol. The findings align with those of [1, 9, 20, 29–32, 59–66](Fig. 4).

To investigate the role of SgTPS-V in *A. thaliana* Columbia-0 (Col-0), we overexpressed *SgTPS-V* in *A. thaliana* integrating Agrobacterium with the pB2GW7-SgTPS-V vector. The *SgTPS-V* expression in successful transformed lines was validated through sqRT-PCR (Fig. 5). The transgenic lines exhibited significantly elevated *SgTPS-V* expression levels versus WT plants, supporting successful introduction of the transgene. Three independent transgenic lines, designated *OE-SgTPS-V-5*, *OE-SgTPS-V-7*, and *OE-SgTPS-V-9*, were selected for terpene analysis. Morphological assessments revealed that these recombinant plants exhibited an accelerated reproductive shoot formation relative to the wild-type (WT) (Fig. 5). In addtion, we used qRT-PCR to elucidate the expression level of *SgTPS-V* gene across different tissues of *S. guaranitica* such as; young leaf, old leaf, stem, flower, bud flower and root, and from our results we found that the *SgTPS-V* gene was highly expression at root tissue, followed by old leaf, young leaf, bud flower, stem and flower. And this previous results are in line with our previous results [1].

These results corroborate previous research by Ali et al., 2017, 2018, 2022a [1, 9, 20], which demonstrated that the upregulation of terpene synthase genes (TPS), such as *SgGPS*,* SoCINS*,* SgFPPS*,* SoTPS6*,* SoLINS*,* SgLINS*,* SoFLDH*,* SgGPS*,* SoSABS*, and *SoNEOD* from *S. guaranitica* and *S. officinalis* in *A. thaliana* and *Nicotiana tabacum*, enhanced vegetative and reproductive development against the WT plants. Prior research indicates that various TPS family members play roles in distinct cellular mechanisms, for example, 1,−8-cineole synthase acting as a monoterpene synthase, Z–γ-bisabolene synthase functioning as a sesquiterpene synthase, rhizathalene synthase (*AtTPS08*) contributing to diterpene biosynthesis, and β-amyrin and thalianol synthases participating in triterpene formation [61–64, 67–71].

Moreover, the co-expression of TPS genes in various cells, tissues, and organs highlights their role in shaping diverse plant phenotypes, reinforcing their significance in plant ontogeny [61–64, 68–72]. Gas-chromatography-mass spectrometry (GC-MS) identified terpenes produced following SgTPS-V overexpression. The resulting transformant lines exhibited distinct peaks corresponding to mono-, sesqui-, and diterpenes, with the percentage peak area indicating the types and relative abundance of these compounds. Terpene identification was carried out using mass spectral libraries, including the Wiley GC/MS and NIST Library, in addition to comparative analysis with wild-type *A. thaliana* extracts.

The GC/MS results (Table 2; Fig. 6) revealed an emergence of two peaks at a retention time of 48.394 and 48.464 min, which were identified as Valencene and (-)-Valencene based on spectral matching with reference databases. However, the comparative analysis of hexane extracts from transgenic and non-transgenic *A. thaliana* revealed distinct, shared, and predominant compounds. The transgenic extract contained unique compounds, along with 46 compounds that were also present in the non-transgenic extract. In the context, the transgenic extract exhibited 50 unique compounds and 11 compounds from them are terpenoids (e.g., All-trans-Geranylgeraniol, Isovaleric acid p-tolyl ester, Cedrenol, β-Carotene, 4,8,13-Duvatriene-1,3-Diol, Epiglobulol, Ledol, isomer 1, trans-caryophyllene oxide, α-Elemol, Isovaleric anhydride and (-)-Myrtenol) (Fig. 6B and supplementary Table 2). The production of Valencene, (-)-Valencene, Valencene (isomer I), (+)-Valencene, Viridiflorine, (-)-Ledol and Patchulane as a sesquiterpene in transgenic *A. thaliana* is consistent with previous reports [1, 48]. Terpene synthases are known to catalyze the synthesis of multiple terpenes, including carene synthases, (±)-linalool synthases, cineole, myrcene, β-amyrin, and terpinolene synthases [25–28, 73–75]. Therefore, *SgTPS-V* is suggested to facilitate valencene and other isoformas biosynthesis via the isoprenoid pathway, a well-characterized mechanism in sesquiterpene production.

In the context, for measurements of enzyme activity the *E. coli* BL21 (DE3) cells expressing SgTPS-V protein using the expression vector pDEST17 were harvested and lysed. Upon incubation with (2E,6E)-farnesyl diphosphate (FPP) as a substrate, SgTPS-V catalyzed the formation of Valencene, and other sesquiterpenes such as; Elemol, Valerena-4,7(11)-diene, Isocaryophillene, cis-Caryophyllene epoxide, Valeranone, and (-)-Globulol that were detected by GC-MS analysis (Fig. 7). The identities of these previous compound peaks were confirmed by comparisons with retention times (R.T; min) and the mass spectra (Molecular Mass; g mol-1) of various libraries. Based on results the major product for SgTPS-V was designated as a Valencene, see Fig. 7. And from our results we found the SgTPS-V belongs to the class of sesquiterpene enzymes which have the ability to catalyze the formation of Valencene with higher levels. At the end, these results confirmed the significant epistatic relationship that was found between the release of Valencene as a sesquiterpene and SgTPS-V gene expression.

In the end, economics studies refer to the great market demand for valencene and its global annual demand exceeds 10,000 kg with market value estimated to grow to 9.2 million dollars by 2033 [76, 77]. Valencene is widely used in the food, beverage, flavor, and a cosmetic fragrance ingredient with extensive applications [76, 77]. In the last decade, valencene supply was primarily relies on extraction from natural sources (e.g., fresh citrus,, woody orange notes, grapefruit peel, other citrus fruits, *Myrica rubra*, *Cyperus rotundus* and *Alpinia oxyphylla Miq*) or chemical synthesis, but the extraction process is affected by some limitations [76, 78]. In Addition, in plant Valencene can also be readily converted to (+)-nootkatone by chemical or biological means, which is not only a commonly used flavor compound in soft drinks but also can be used as mosquito repellant [76, 78]. Several studies used the valencene compound level as an indicator of good quality of orange fruit, orange oil, fruit maturation, fruit development period and the level of the citrus greening disease [79, 80]. Furthermore, in plant valencene synthase can be affected by the plant hormone stimuli such as ethylene, development stages, irrigation conditions, and synthesis of other volatile terpenoid products [81, 82]. Moreover, the increased demand for valencene has attracted considerable attention from researchers to develop ways for increasing Valencene production by transgenic plants or by novel microbial cell factories for more efficient and sustainable production modes. As a result, this study provides a clearer vision through study of the function of the *SgTPS-V* gene from *S. guaranitica* as a new source of valencene synthese.

## Conclusion

In this study, we successfully cloned and characterized *SgTPS-V*, a valencene synthase gene from *S. guaranitica*, a medicinal plant with significant pharmacological properties. Overexpression of *SgTPS-V* in *A. thaliana* accelerated flowering in transgenic lines (OE-*SgTPS-V*−5, OE-*SgTPS-V*−7, and OE-*SgTPS-V*−9) and led to increased valencene production, confirming that *A. thaliana* can synthesize valencene via the mevalonate (MVK) pathway of sesquiterpene biosynthesis. Computational analysis indicated that *SgTPS-V* displayed significant expression levels across multiple tissues and its product can be found in plastids and mitochondria, underscoring its role in terpene biosynthesis. These findings not only show that *A. thaliana* is a robust model for functional studies of sesquiterpene synthases but also provide a foundation for enhancing ornamental properties in the target species via synthetic biology.

## Supplementary Information

Supplementary Material 1.

Supplementary Material 2.

## Data Availability

All data generated or analyzed during this study are included in this published article and its supplementary information files. The datasets used and/or analyzed during the current study are available from the corresponding author on reasonable request. GenBank accession number: Salvia guaranitica Valencene Synthase (SgTPS-V, GenBank: KX893974.1) https://www.ncbi.nlm.nih.gov/nuccore/KX893974.1.

## References

[1] Ali M, Hussain RM, Rehman NU, She G, Li P, Wan X, Guo L, Zhao J. De Novo transcriptome sequencing and metabolite profiling analyses reveal the complex metabolic genes involved in the terpenoid biosynthesis in blue Anise Sage (Salvia guaranitica L). DNA Res. 2018;25:597–617. 10.1093/dnares/dsy028. 30188980 10.1093/dnares/dsy028 PMC6289780

[2] El-ramah FA, Mohammed A, Esraa AE, Manal KA. Molecular cloning and characterization of beta-amyrin synthase (SoAMYS) gene from salvia officinalis plant. Egypt J Desert Res. 2022;72(1):27–45. 10.21608/EJDR.2022.122501.1099.

[3] Kamatoua GPP, Makungab NP, Ramogolab WPN, Viljoen AM. South African salvia species: A review of biological activities and phytochemistry. J Ethnopharmacol. 200;119:664–72. 10.1016/j.jep.2008.06.030 18640254

[4] Takano A, Okada H. Phylogenetic relationships among subgenera, species, and varieties of Japanese salvia L. (Lamiaceae). J Plant Res.2011; (124): 245–52. 10.1007/s10265-010-0367-9 20628783

[5] Fateme AM, Mohammad HF, Abdolhossein R, Ali Z, Maryam S. Volatile constituents of *Salvia compressa* and *Logochilus macranthus*, two labiatae herbs growing wild in Iran. Res J Recent Sci 2013; (2): 66–8.

[6] Li D, Shao F, Lu S. Identification and characterization of mRNA-like noncoding RNAs in salvia miltiorrhiza. Planta. 2015;241:1131–43. 25601000 10.1007/s00425-015-2246-z

[7] Wang B, Sun W, Li Q, Li Y, Luo H, Song J, Sun C, Qian J, Zhu Y, Hayward A, Xu H, Chen S. Genome-wide identification of phenolic acid biosynthetic genes in *Salvia miltiorrhiza*. Planta. 2015a;241(3):711–25. 25471478 10.1007/s00425-014-2212-1

[8] Zhenqing B, Wenrui L, Yanyan J, Zhiyong Y, Jie J, Wenli H, Pengguo X, Zongsuo L. The ethylene response factor *SmERF6* co-regulates the transcription of *SmCPS1* and *SmKSL1* and is involved in Tanshinone biosynthesis in *Salvia miltiorrhiza* hairy roots. Planta. 2018; (248): 243–55. 10.1007/s00425-018-2884-z 29704055

[9] Mohammed A, Dikhnah A, Abeer MA, Naeema AE, Doaa BED. Cloning and characterization of 1, 8-cineole synthase (SgCINS) gene from the leaves of salvia guaranitica plant. Front Plant Sci. 2022a;13:1–15. 10.3389/fpls.2022.8694. 10.3389/fpls.2022.869432 PMC9051517 35498676

[10] Ali M. Cloning, molecular characterization and functional analysis of the cis-muuroladiene synthase (SgCMS) gene from leaves of salvia guaranitica plant. Egypt J Desert Res. 2023;73(1):239–63. 10.21608/ejdr.2023.209304.1142.

[11] Durairaj J, Di G, Bouwmeester A, de Ridder HJ, Beekwilder D, van Dijk J. An analysis of characterized plant sesquiterpene synthases. Phytochemistry. 2019;158:157–65. 10.1016/j.phytochem.2018.10.020. 30446165 10.1016/j.phytochem.2018.10.020

[12] Singh B, Sharma RA. Plant terpenes: defense responses, phylogenetic analysis, regulation and clinical applications. 3 Biotech. 2015;5(2):129–51. 10.1007/s13205-014-0220-2. 28324581 10.1007/s13205-014-0220-2 PMC4362742

[13] Evidente A, Kornienko A, Lefranc F, Cimmino A, Dasari R, Evidente M, Mathieu V, Kiss R. Sesterterpenoids with anticancer activity. Curr Med Chem. 2015;22(30):3502–22. 10.2174/0929867322666150821101047. 26295461 10.2174/0929867322666150821101047 PMC4955362

[14] Piechulla B, Bartelt R, Brosemann A, Effmert U, Bouwmeester H, HippaufF, Brandt W. The α-Terpineol to 1, 8-Cineole cyclization reaction of tobacco terpene synthases. Plant Physiol. 2016;172(4):2120–31. 10.1104/pp.16.01378. 27729471 10.1104/pp.16.01378 PMC5129724

[15] Ker DS, Pang SL, Othman NF, Kumaran S, Tan EF, Krishnan T, Chan KG, Othman R, Hassan M, Ng CL. Purification and biochemical characterization of Recombinant *Persicaria minor β*-sesquiphellandrene synthase. PeerJ. 2017;5:e2961. 10.7717/peerj.2961. 28265494 10.7717/peerj.2961 PMC5333544

[16] Chen H, Köllner TG, Li G, Wei G, Chen X, Zeng D, Qian Q, Chen F. Combinatorial evolution of a terpene synthase gene cluster explains terpene variations in *Oryza*. Plant Physiol. 2020;182(1):480–92. 10.1104/pp.19.00948. 31712306 10.1104/pp.19.00948 PMC6945850

[17] Ali M, Miao L, Hou Q, Darwish DB, Alrdahe SS, Ali A, Benedito VA, Tadege M, Wang X, Zhao J. Overexpression of terpenoid biosynthesis genes from garden Sage (*Salvia officinalis*) modulates rhizobia interaction and nodulation in soybean. Front Plant Sci. 2021;12: 783269. 35003167 10.3389/fpls.2021.783269 PMC8733304

[18] Gershenzon J, Kreish W. Biochemistry of terpenoids: monoterpenes, sesquiterpenes, diterpenes, sterols, cardiac glycosides and steroid saponins. In: Wink M, editor. Biochemistry of plant secondary metabolism. Florida: CRC; 1999. pp. 222–99.

[19] İsmail P, Merve S. Partial cloning and identification of terpene synthase-6 gene (Tps-6) in an aromatic plant *Origanum onites* l. Trak Univ J Nat Sci. 2017;18(2):137–42.

[20] Ali M, Li P, She G, Chen D, Wan X, Zhao J. Transcriptome and metabolite analyses reveal the complex metabolic genes involved in volatile terpenoid biosynthesis in garden Sage (Salvia officinalis). Sci Rep. 2017;7:16074. 10.1038/s41598-017-15478-3. 29167468 10.1038/s41598-017-15478-3 PMC5700130

[21] Chang Y, Wang M, Li J, Lu S. Transcriptomic analysis reveals potential genes involved in Tanshinone biosynthesis in *Salvia miltiorrhiza*. Sci Rep. 2019;9(1):14929. 10.1038/s41598-019-51535-9. 31624328 10.1038/s41598-019-51535-9 PMC6797793

[22] Lu LL, Zhang YX, Yang YF. Integrative transcriptomic and metabolomic analyses unveil Tanshinone biosynthesis in *Salvia miltiorrhiza* root under N starvation stress. PLoS ONE. 2022;17(8):e0273495. 10.1371/journal.pone.0273495. 36006940 10.1371/journal.pone.0273495 PMC9409544

[23] McAndrew RP, Peralta-Yahya PP, DeGiovanni A, Pereira JH, Hadi MZ, Keasling JD, Adams PD. Structure of a three-domain sesquiterpene synthase: a prospective target for advanced biofuels production. Struct (London England:1993). 2011;19(12):1876–84. 10.1016/j.str.2011.09.013. 10.1016/j.str.2011.09.013 22153510

[24] Shi M, Zhou W, Zhang J, Huang S, Wang H, Kai G. Methyl jasmonate induction of Tanshinone biosynthesis in *Salvia miltiorrhiza* hairy roots is mediated by jasmonate Zim-Domain repressor proteins. Sci Rep. 2016;6:20919. 10.1038/srep20919. 26875847 10.1038/srep20919 PMC4753458

[25] Yoko I, David RG, Eyal F, Efraim L, Eran P. Characterization of geraniol synthase from the peltate glands of sweet Basil. Plant Physiol. 2004; (134): 370–9. 10.1104/pp.103.032946 PMC316316 14657409

[26] Shimada T, Endo T, Fujii H, Hara M, Omura M. Isolation and characterization of (E)-beta-ocimene and 1, 8 cineole synthases in *Citrus Unshiu* Marc. Plant Sci. 2005;(168):987–95.

[27] Abbas F, Yanguo K, Rangcai Y, Yanping F. Functional characterization and expression analysis of two terpene synthases involved in floral scent formation in *Lilium*. ‘Siberia ’ Planta. 2019;(249):71–93. 10.1007/s00425-018-3006-7 30218384

[28] Lucker J, El Tamer MK, Schwab W, Verstappen FW, van der Plas LH, Bouwmeester HJ, Verhoeven HA. Monoterpene biosynthesis in lemon (*Citrus limon*). cDNA isolation and functional analysis of four monoterpene synthases. Eur J Biochem. 2002; (269):3160–71. 10.1046/j.1432-1033.2002.02985.x 12084056

[29] Mohammed A, Elsayed N, Walaa AR, Mohamed E, Mokhtar SR, Ahmed GMS-E, Mohamed ASE-Z, Ahmed HMH, Mingquan G, Guang-Wan H, Shengwei W, Fatma AA, Mohamed HA, Qing-Feng W. Molecular characterization of a novel NAD+-dependent Farnesol dehydrogenase SoFLDH gene involved in sesquiterpenoid synthases from salvia officinalis. PLoS ONE. 2022b;176:1–19. 10.1371/journal.pone.0269045. 10.1371/journal.pone.0269045 PMC9165828 35657794

[30] Mohammed A, Miao L, Soudy FA, et al. Overexpression of terpenoid biosynthesis genes modifies root growth and nodulation in soybean (*Glycine max*). Cells. 2022c;11(17):2622. 10.3390/cells11172622. 36078031 10.3390/cells11172622 PMC9454526

[31] Mohammed A, El-ramah FA, Esraa AE, Mohamed NSS, Mokhtar SR. Cloning and characterization of terpene synthase 3 (SoTPS3) gene from leaves of garden Sage (Salvia officinalis). Jordan J Biol Sci. 2023;673–85. 10.54319/jjbs/160413.

[32] Dereeper A, Guignon V, Blanc G, Audic S, Buffet S, Chevenet F, Dufayard JF, Guindon S, Lefort V, Lescot M, Claverie JM, Gascuel O. Phylogeny.fr: robust phylogenetic analysis for the non-specialist. Nucleic Acids Res.2008; (36):W465–9. 10.1093/nar/gkn180 PMC2447785 18424797

[33] Mehmood N, Yuan Y, Ali M, Ali M, Iftikhar J, Cheng C, Lyu M, Wu B. Early transcriptional response of terpenoid metabolism to Colletotrichum gloeosporioides in a resistant wild strawberry Fragaria nilgerrensis. Phytochemistry. 2021;181:112590. 33232864 10.1016/j.phytochem.2020.112590

[34] Hussain RM, Ali M, Feng X, Li X. The essence of NAC gene family to the cultivation of drought-resistant soybean (*Glycine max* L. Merr.) cultivars. BMC Plant Biol. 2017;17(1):55. 28241800 10.1186/s12870-017-1001-y PMC5330122

[35] Darwish DBE, Mohammed A, Abdelkawy AM, Zayed M, Alatawy M, Nagah A. Constitutive overexpression of GsIMaT2 gene from wild soybean enhances rhizobia interaction and increase nodulation in soybean (Glycine max). BMC Plant Biol. 2022;22(1):431. 10.1186/s12870-022-03811-6. 36076165 10.1186/s12870-022-03811-6 PMC9461152

[36] Reem MH, Mohammed A, Xing F, Xia L. The essence of NAC gene family to the cultivation of drought-resistant soybean (Glycine max L. Merr.) cultivars. BMC Plant Biol. 2017;17:55. 28241800 10.1186/s12870-017-1001-y PMC5330122

[37] Rehman N, Mohammed A, Ahmad M, Liang G, Zhao J. Strigolactones promote rhizhobia interaction and increase nodulation in soybean (Glycine max). Microbial Patho. 2017, 2; 114:420–430. 10.1016/j.micpath. 2017.11.049. 10.1016/j.micpath.2017.11.049 29191709

[38] Abbas ZK, Al-Huqail AA, Abdel Kawy AH, Abdulhai RA, Albalawi DA, AlShaqhaa MA, Alsubeie MS, Darwish DBE, Abdelhameed AA, Soudy FA, Makki RM, Aljabri M, Al-Sulami N, Ali M, Zayed M. Harnessing de Novo transcriptome sequencing to identify and characterize genes regulating carbohydrate biosynthesis pathways in salvia guaranitica L. Front. Plant Sci. 2024;15:1467432. 10.3389/fpls.2024.146743. 10.3389/fpls.2024.1467432 PMC11464306 39391775

[39] Mohammed A, Fatma MOA, Ahmed AA, Fathia AS, Doaa BEDa ESZIM. Rasha.MAk, Karima MEl, Aesha H AK. Physiological and transcriptomic evaluation of salt tolerance in Egyptian tomato landraces at the seedling stage. BMC Plant Biol. 2025;15:112–35. 10.1186/s12870-025-06358-4 PMC12013233 40259234

[40] Abd El-Wahab M, Toaima W, Hamed E. Effect of different planting locations in Egypt on salvia fruticosa mill. Plants. Egypt J Desert Res 2015; 65(2):291–307. doi: 10.21608/ejdr.2015.5955.

[41] Khater R. Evaluating the productivity of salvia officinalis, l. plants using of fertilizers and spraying with vitamins. Egypt J Desert Res. 2022;72(1):47–71. 10.21608/ejdr.2022.115098.1097.

[42] Toaima W. Effect of organic fertilization and active dry yeast on productivity of three-lobed Sage (Salvia fruticosa mill.) plants under Siwa Oasis conditions. Egypt J Desert Res. 2014;64(1):153–66. 10.21608/ejdr.2014.5815.

[43] Gao F, Liu B, Li M, Gao X, Fang Q, Liu C, Ding H, Wang L, Gao X. Identification and characterization of terpene synthase genes accounting for volatile terpene emissions in flowers of freesia x hybrida. J Exp Bot. 2018;69(18):4249–65. 10.1093/jxb/ery224. 29901784 10.1093/jxb/ery224 PMC6093421

[44] Ahmad MZ, Zhang Y, Zeng X, Li P, Wang X, Benedito VA, Zhao J. (2021). Isoflavone malonyl-CoA acyltransferase GmMaT2 is involved in nodulation of soybean by modifying synthesis and secretion of isoflavones. J Exp Bot. 2021, 72(4):1349–1369. 10.1093/jxb/eraa511 10.1093/jxb/eraa511 33130852

[45] Ahmad MZ, Li P, Wang J, Rehman NU, Zhao J. 2017. Isoflavone malonyltransferases GmIMaT1 and GmIMaT3 differently modify isoflavone glucosides in soybean (Glycine max) under various stresses. Front. Plant Sci. 2017, 8, 735. 10.3389/fpls.2017.00735 10.3389/fpls.2017.00735 PMC5433297 28559900

[46] Yahyaa M, Matsuba Y, Brandt W, Doron-Faigenboim A, Bar E, McClain A, Davidovich-Rikanati R, Lewinsohn E, Pichersky E, Ibdah M, Identification. Functional Characterization, and evolution of terpene synthases from a basal Dicot. Plant Physiol. 2015;169(3):1683–97. 10.1104/pp.15.00930. 26157114 10.1104/pp.15.00930 PMC4634067

[47] Mohammed A, Aisha MA, Doaa BED, Hanan AA, Dikhnah A, Hadba A-A, Fathia AS. Changes in metabolite profiling and expression levels of key genes involved in the terpenoid biosynthesis pathway in garden Sage (Salvia officinalis) under the effect of hydrazine hydrate. Metabolites. 2023b;13(807):1–15. 10.3390/metabo1307080. 10.3390/metabo13070807 PMC10385164 37512514

[48] Su-Fang E, Zeti-Azura M, Roohaida O, Noor AS, Ismanizan I, Zamri Z. Functional characterization of sesquiterpene synthase from *Polygonum minus*. Sci World J. 2014. 10.1155/2014/840592. 10.1155/2014/840592 PMC3942395 24678279

[49] Degenhardt J, Köllner T, Gershenzon J. Monoterpene and sesquiterpene synthases and the origin of terpene skeletal diversity in plants. Phytochemistry. 2009;70:1621–37. 19793600 10.1016/j.phytochem.2009.07.030

[50] Abdelhameed AA, Eissa MA, El-kholy RI, Darwish DBE, Abeed AHA, Soudy FA, Alyamani AA, Abdelmigid HM, Morsi MM, Zhao J, Mohammed A, Muhammad Z. Molecular cloning and expression analysis of geranyllinalool synthase gene (SgGES) from salvia guaranitica plants. Horticulturae. 2024;10:668. 10.3390/horticulturae10070668.

[51] Esraa AE, Mohammed A, El-Ramah FA, Manal KA. Molecular cloning and characterization of terpene synthase 4 (SgTPS4) gene from salvia guaranitica plant. Egypt J Genet Cytol, 202; 51(1):1–15https://journal.esg.net.eg/index.php/EJGC/article/view/352

[52] Nasir M, Yuan Y, Mohammed A, Muhammad A, Junaid I, Chunzhen C, Meiling L, Binghua W. Early transcriptional response of terpenoid metabolism to Colletotrichum gloeosporioides in a resistant wild strawberry Fragaria nilgerrensis. Phytochemistry. 2021;181:112590, 1–11. 10.1016/j.phytochem.2020.112590. 10.1016/j.phytochem.2020.112590 33232864

[53] Lima AS, Jette S, Brigitte L, Johannes N, Jose´ GB, Figueiredo AC, Luis GP, Jo¨rg D, Helena T. Genomic characterization, molecular cloning and expression analysis of two terpene synthases from *Thymus caespititius* (Lamiaceae). Planta. 2013; (238):191–204. 10.1007/s00425-013-1884-2 23624978

[54] López-Gallego F, Wawrzyn GT, Schmidt-Dannert C. Selectivity of fungal sesquiterpene synthases: role of the active site’s H-1α loop in catalysis. Appl Environ Microbiol. 2010;76:7723–33. 20889795 10.1128/AEM.01811-10 PMC2988597

[55] Christianson DW. Structural biology and chemistry of the terpenoid cyclases. Chem Rev. 2006;106:8. 10.1021/cr050286w 16895335

[56] Davis EM, Croteau R. Cyclization enzymes in the biosynthesis of monoterpenes, sesquiterpenes, and diterpenes. In: Vederas JC, Leeper FJ, editors. Biosynthesis: aromatic Polyketides, Isoprenoids, alkaloids. Eds: Springer, Berlin, Germany; 2000. pp. 53–95.

[57] Whittington DA, Wise ML, Urbansky M, Coates RM, Croteau RB, Christianson DW. Bornyl diphosphate synthase: structure and strategy for carbocation manipulation by a terpenoid cyclase. Proc Natl Acad SciUSA. 2002; (99):15375–80. 10.1073/pnas.232591099 PMC137724 12432096

[58] Hyatt DC, Youn B, Zhao Y, Santhamma B, Coates RM, Croteau RB, Kang C. Structure of limonene synthase, a simple model for terpenoid cyclase catalysis. Proc Natl Acad Sci.2007; (104):5360–5. 10.1073/pnas.0700915104 PMC1838495 17372193

[59] Taniguchi S, Miyoshi S, Tamaoki D, Yamada S, Tanaka K, Uji Y, Tanaka S, Akimitsu K, Gomi K. Isolation of jasmonate-induced sesquiterpene synthase of rice: product of which has an antifungal activity against Magnaporthe oryzae. J Plant Physiol. 2014;171:625–32. 24709155 10.1016/j.jplph.2014.01.007

[60] Wang B, Wei S, Qiushi L, Ying L, Hongmei L, Jingyuan S, Chao S, Jun Q, Yingjie Z, Alice H, Haibin X, Shilin C. Genome-wide identification of phenolic acid biosynthetic genes in *Salvia miltiorrhiza*. Planta 2015; (241):711–25. 10.1007/s00425-014-2212-1 25471478

[61] Wang Q, Jia M, Huh JH, Muchlinski A, Peters RJ, Tholl D. Identification of a dolabellane type diterpene synthase Andother Root-Expressed diterpene synthases in Arabidopsis. Front Plant Sci. 2016;7:1761. 27933080 10.3389/fpls.2016.01761 PMC5122590

[62] Chen X, Chen H, Yuan JS, Ko¨llner TG, Chen Y, Guo Y, et al. The rice terpene synthase gene OsTPS19 functions as an (S)-limonene synthase in planta, and its overexpression leads to enhanced resistance to the blast fungus *Magnaporthe oryzae*. Plant Biotechnol J. 2018;16(10):1778–87. 29509987 10.1111/pbi.12914 PMC6131416

[63] Chen F, Ro DK, Petri J, Gershenzon J, Bohlmann J, Pichersky E, Tholl D. Characterization of a root-specific Arabidopsis terpene synthase responsible for the formation of the volatile monoterpene 1, 8-cineole. Plant Physiol. 2004; (135):1956–66. 10.1104/pp.104.044388 PMC520767 15299125

[64] Chen F, Tholl D, Bohlmann J, Pichersky E. The family of terpene synthases in plants: A mid-size family of genes for specialized metabolism that is highly diversified throughout the Kingdom. Plant J. 2011;(66): 212–29. 10.1111/j.1365-313X.2011.04520.x 21443633

[65] Makhadmeh I, Albalasmeh AA, Ali M, Thabet SG, Darabseh WA, Jaradat S, Alqudah AM. Molecular characterization of tomato (*Solanum lycopersicum* L.) accessions under drought stress. Horticulturae. 2022b;8(7):600.

[66] Makhadmeh IM, Thabet SG, Ali M, Alabbadi B, Albalasmeh A, Alqudah AM. Exploring genetic variation among Jordanian solanum Lycopersicon L. landraces and their performance under salt stress using SSR markers. J Genet Eng Biotechnol. 2022a;20(1):45. 35275332 10.1186/s43141-022-00327-2 PMC8917245

[67] Ro D-K, Ehlting J, Keeling CI, Lin R, Mattheus N, Bohlmann J. Microarray expression profiling and functional characterization of AtTPS genes: duplicated *Arabidopsis Thaliana* sesquiterpene synthase genes At4g13280 and At4g13300 encode root-specific and wound-inducible (Z)-γ-bisabolene synthases. Arch Biochem Biophys. 2006;(448):104–16. 10.1016/j.abb.2005.09.019 16297850

[68] Kampranis SC, Ioannidis D, Purvis A, Mahrez W, Ninga E, Katerelos NA, Anssour S, Dunwell JM, Degenhardt J, Makris AM, Goodenough PW, Johnson CB. Rational conversion of substrate and product specificity in a salvia monoterpene synthase: structural insights into the evolution of terpene synthase function. Plant Cell. 2007;19(6):1994–2005. 17557809 10.1105/tpc.106.047779 PMC1955729

[69] Field B, Fiston-Lavier AS, Kemen A, Geisler K, Quesneville H, Osbourn AE. Formation of plant metabolic gene clusterswithin dynamic chromosomal regions. Proc. Natl. Acad. Sci. USA.2011; (108):16116–16121. 10.1073/pnas.1109273108 PMC3179108 21876149

[70] Field B, Osbourn AE. Metabolic diversification–independent assembly of operon-like gene clusters in different plants. Science. 2008;320:543–7. 18356490 10.1126/science.1154990

[71] Vaughan MM, Wang Q, Webster FX, Kiemle D, Hong YJ, Tantillo DJ, Coates RM, Wray AT, Askew W, O’Donnell C, Tokuhisa JG, Tholl D. Formation of the unusual semivolatile diterpene rhizathalene by the Arabidopsis class I terpene synthaseTPS08 in the root stele is involved in defense against belowground herbivory. Plant Cell 2013;(25): 1108–25. 10.1105/tpc.112.100057 PMC3634680 23512856

[72] Croteau R, Felton M, Karp F, Kjonaas R. Relationship of Camphor biosynthesis to leaf development in Sage salvia officinalis. Plant Physiol. 1981;67:820–4. 16661761 10.1104/pp.67.4.820 PMC425779

[73] Fahnrich A, Krause K, Piechulla B. Product variability of the ‘cineole cassette’ monoterpene synthases of related Nicotiana species. Mol Plant. 2011;4:965–84. 21527560 10.1093/mp/ssr021

[74] XI, LR, XL, D-YX. Overexpression of a synthetic insect–plant Geranyl pyrophosphate synthase gene in camelina sativa alters plant growth and terpene biosynthesis. Planta. 2016;244:215–30. 10.1007/s00425-016-2504-8 27023458

[75] Faldt J, Martin D, Miller B, Rawat S, Bohlmann J. Traumatic resin defense in Norway Spruce (Picea abies): Methyl jasmonate-induced terpene synthase gene expression, and cDNA cloning and functional characterization of (+)-3-carene synthase. Plant Mol Biol. 2003;51:119–33. 12602896 10.1023/a:1020714403780

[76] Song Y, Liu H, Quax WJ, Zhang Z, Chen Y, Yang P, Cui Y, Shi Q, Xie X. Application of Valencene and prospects for its production in engineered microorganisms. Front Microbiol. 2024;151444099. 10.3389/fmicb.2024.1444099. 10.3389/fmicb.2024.1444099 PMC11335630 39171255

[77] Li Z, Li W, Gao X, Yao W, Zhu Z, Luo X, ZhangY Yuan J. Systematic engineering to enhance Valencene production in rhodobacter sphaeroides. Bioresources Bioprocess. 2025;12(1):100. 10.1186/s40643-025-00942-0. 10.1186/s40643-025-00942-0 PMC12449282 40973815

[78] Dietsch M, Behle A, Westhoff P, Axmann IM. Metabolic engineering of synechocystis sp. PCC 6803 for the photoproduction of the sesquiterpene Valencene. Metab Eng Commun. 2021;13e00178. 10.1016/j.mec.2021.e00178. 10.1016/j.mec.2021.e00178 PMC8382996 34466381

[79] Elston A, Lin JM, Rouseff R. Determination of the role of Valencene in orange oil as a direct contributor to aroma quality. Flavour Frag J. 2005;20381–6. 10.1002/ffj.1578.

[80] Benelli G, Caruso G, Giunti G, Cuzzola A, Saba A, Raffaelli A, et al. Changes in Olive oil volatile organic compounds induced by water status and light environment in canopies of Olea Europaea L. trees. J Sci Food Agric. 2015;952473–81. 10.1002/jsfa.6977. 10.1002/jsfa.6977 25355375

[81] Binder D, Frohwitter J, Mahr R, Bier C, Grunberger A, Loeschcke A, et al. Light-controlled cell factories: employing photocaged isopropyl-beta-d-Thiogalactopyranoside for light-mediated optimization of Lac promoter-based gene expression and (+)-Valencene biosynthesis in Corynebacterium glutamicum. Appl Environ Microbiol. 2016;826141–9. 10.1128/AEM.01457-16. 10.1128/AEM.01457-16 PMC5068161 27520809

[82] Shen SL, Yin XR, Zhang B, Xie XL, Jiang Q, Grierson D, et al. CitAP2.10 activation of the terpene synthase CsTPS1 is associated with the synthesis of (+)-valencene in ‘Newhall’ orange. J Exp Bot. 2016;674105–15. 10.1093/jxb/erw189. 10.1093/jxb/erw189 PMC5301923 27194737

