# Supplementary material for: Molecular insights into Valencene synthase (SgTPS-V) and its role in sesquiterpenoid biosynthesis
Source: BMC Plant Biol. 2026 Feb 2;26:379. doi: 10.1186/s12870-026-08095-8 (PMC12930907; doi:10.1186/s12870-026-08095-8)
Supplement: Supplementary file 2 — Supplementary Material 2. [file 12870_2026_8095_MOESM2_ESM.pptx]

## Slide 1
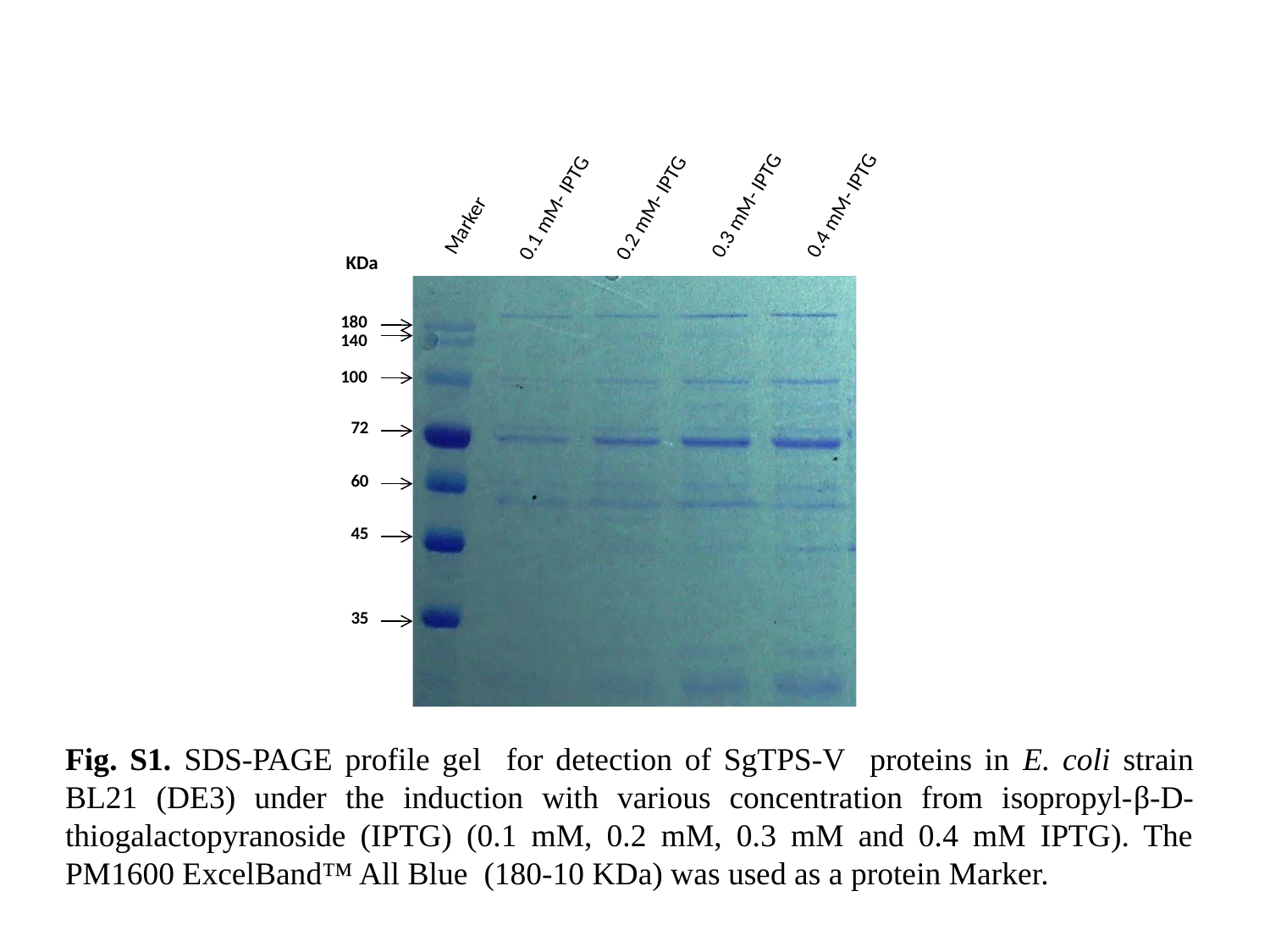

Marker
 0.3 mM- IPTG
 0.4 mM- IPTG
 0.1 mM- IPTG
 0.2 mM- IPTG
KDa
180
140
100
72
60
45
35
Fig. S1. SDS-PAGE profile gel for detection of SgTPS-V proteins in E. coli strain BL21 (DE3) under the induction with various concentration from isopropyl-β-D-thiogalactopyranoside (IPTG) (0.1 mM, 0.2 mM, 0.3 mM and 0.4 mM IPTG). The PM1600 ExcelBand™ All Blue (180-10 KDa) was used as a protein Marker.
